# Editorial: Insights in Gastrointestinal and Hepatic Pharmacology: 2021

**DOI:** 10.3389/fphar.2022.890212

**Published:** 2022-04-05

**Authors:** Ralf Weiskirchen, Angelo A. Izzo

**Affiliations:** ^1^ Institute of Molecular Pathobiochemistry, Experimental Gene Therapy and Clinical Chemistry (IFMPEGKC), RWTH University Hospital Aachen, Aachen, Germany; ^2^ Department of Pharmacy, School of Medicine and Surgery, University of Naples Federico II, Naples, Italy

**Keywords:** therapy, experimental models, clinical evaluation, liver, gastrointestinal tract, drugs, intervention, clinical trial

## Introduction

There are many diseases of the gastrointestinal tract and the liver that affect patients’ quality of life. Since gastrointestinal diseases can affect the gastrointestinal tract from the mouth to the anus, functional and structural impairments can vary widely. Common problems include abdominal discomfort, upper or lower gastrointestinal bleeding, constipation, loss of appetite, unintentional weight loss, nausea and vomiting, fatigue, as well as constipation and/or diarrhea. In addition, when becoming chronically, they can result in chronic symptoms associated with pain, dyspepsia and altered bowel habitat. Based on the versatility of these diseases, it is obvious that there must be an abundance of genetic and epigenetic factors contributing to the progression or outcome of respective illnesses. Current therapies and treatment options encompass dietary, pharmacologic, recommendations for lifestyle changes, and complementary medical treatment. Management of patients could also consider cognitive behavioral therapy and psychotropic drug therapy. Nevertheless, therapy is often challenging requiring personalized forms of treatment. Recent years of research and clinical observations have further shown that there are numerous mechanisms by which genetic and epigenetic factors contribute to gastrointestinal and hepatic disease initiation and progression providing a large list of both diagnostic and therapeutic targets. However, despite the recent progress made, the etiology of these diseases are still incompletely understood and studies providing a better understanding of the pathogenesis and the factors driving the underlying processes are urgently needed.

This Research Topic covers a wide range of aspects showing that the field of gastrointestinal and hepatic diseases has made conceptual advances that hopefully will end in novel treatment options in the near future.

## New Insights in Gastrointestinal Diseases

Geesala et al. discuss the pathophysiolocal significance of excessive mechanical stress in inflammatory conditions and functional bowel disorders. The review provides data showing that mechanical stress can profoundly alter gene expression in the GI tract resulting in induced expression of pro-inflammatory molecules, pain mediators, pro-fibrotic genes and growth factors in obstructive, inflammatory, and functional bowel disorders. As such excess mechanical stress is a key factor critically contributing to pathophysiological changes in the GI tract, potentially offering new therapeutic targets for the management of GI disorders.

A thematically related review by Singh et al. highlights current treatment options and novel therapeutic insights for gastrointestinal dysmotility and functional gastrointestinal disorders. The comprehensive overview discusses the heterogeneity of respective diseases, currently available treatment options for targeting the pathophysiological mechanisms, limitations in effectiveness and safety profile of present drugs, and therapeutic clues for clinical care and management of patients suffering from various functional gastrointestinal disorders. The review demonstrates that there is still a lack in understanding the pathophysiology underlying functional and motility gastrointestinal disorders that may be the consequence of the heterogeneous and complex nature of gut-brain interactions.

Sun et al. tested the therapeutic impact of a novel traditional herbal Chinese medicine prescription, namely Qingchang Wenzhong Decoction (QCWZD), on the recovery phase of colitis in the model of dextran sulfate sodium (DSS)-induced colitis in mice. The authors could demonstrate that QCWZD orally administrated at a dosage of 1.8 g/kg body weight provoked faster recovery from epithelial injury, reduction of mucosal inflammation, and attenuation of intestinal dysbiosis. Fecal microbiome transplantation and antibiotic treatment further revealed that the observed effects were gut microbiota dependent. Collectively, these data suggest that QCWZD accelerates intestinal mucosal healing through the modulation of dysregulated gut microbiota.

The meta-analysis by Huang et al. systematically analyzed the protective effects of perioperative usage of the selective α_2_-adrenoceptor agonist dexmedetomidine in reducing Ischemia-reperfusion (IR) injury induced by hepatotectomy. Based on findings reported in eight randomized controlled trials with a total of 468 participants dexmedetomidine is associated with a systematic decrease in inflammatory response and oxidative stress resulting in protective effects against hepatic IR injury after hepatectomy. This was correlated with reduced concentrations of urea nitrogen, creatinine and serum diamine oxidase, suggesting that the drug also prevents renal and intestinal injury in the setting of IR injury induced by hepatotectomy.

The study by Li et al. investigated the effects of exogenous hydrogen sulfide in the paraventricular nucleus of the hypothalamus on the gastric function of rats. The authors could show that microinjection of sodium hydrogen sulfide (NaHS) physiologically acting as a hydrogen sulfide (H_2_S) donor inhibits gastric motility and promotes gastric acid secretion by modulating activity of the paraventricular nucleus. Interestingly, inhibitors that target the N-methyl-D-aspartic acid receptor or the NF-κB signaling pathway eliminated these effects suggesting that the axis between the paraventricular nucleus and gastric functions is controlled or modulated by respective pathways.

Li et al. contributed a review article in which the biological significance of gut microbiota-derived impact indoles and its derivatives are reviewed in the context of gastrointestinal and hepatic diseases. In their article the general metabolism of indole, related pathways and enzymes, its pathogenetic relevance, and potential therapeutic activities of this substance are discussed. The presented overview suggests that a better understanding of indole biology in the pathogenesis of pathological conditions will help to assist on developing novel individualized bacteriotherapies.

The meta-analyis performed by Morino et al. evaluated the efficacy and safety of proton pump inhibitor-amoxicllin-clarithromycin (PPI-AMPC-CAM) triple therapy for eradication of *Helicobacter pylori* with special emphasis on different cytochrome P450 2C19 genotypes. In their study, the cure rates of PPI-AMPC-CAM *H. pylori* eradication of 25 RCTs with a total of 5,318 patients were systematically analyzed. The study revealed that the cure rates of the PPI-AMPC-CAM eradication therapy significantly differ among CYP2C19 genotypes. While the percentage of eradiation in patients with a poor metabolizer CYP2C19 genotype is rather high (>85%), the cure rate in subjects with an extensive metabolizer genotype was rather low. Therefore, the authors suggested that CYP2C19 genotyping before initiation of eradication therapy will be helpful to achieve higher eradication rates.

Orts et al. prospectively analyzed the serum concentrations of anti-TNF in trough samples at a steady state in Crohn’s disease patients that were either treated with infliximab (n = 62) or adalimumab (n = 49). Interestingly, in both collectives the patients showed different systemic concentrations of cytokines that were directly correlated to the drug concentrations reached in the serum. The differences were further associated with clinical differences. In particular, the hospitalization rate in the adalimumab groups was inversely correlated to the reached effective drug concentration, suggesting that in future studies it will be necessary to consider not only the drug and dose administered, but also the final systemic drug concentration in each Crohn’s disease patient.

The Mini Review by Tolaymat et al. summarizes recent data on the pharmacology and availability of agonists and antagonists of muscarinic receptors for research and therapeutics. Presently, the five different subgroups of these guanine nucleotide protein-coupled receptors (M_1_R-M_5_R) are supposed to have divergent and opposing effects on the progression of gastric, pancreatic, and colon cancer, and liver injury and fibrosis. The discussed data demonstrates that potential drugs selectively targeting either M1R or M3R molecules with dual functionality acting as an M1R agonist and M3R antagonist should have great therapeutic potential. However, presently the development of such drugs is somewhat limited by the complexity of receptor functions and responses, the extensive sequence homology between the different RM subtypes hampering the creation of selective drugs, and current gaps in general knowledge of the remarkable diverse activities of these receptors in the pathogenesis of gastrointestinal and liver diseases.

Traini et al. investigated the impact of chronic exposure to cigarette smoke and pathogenesis of inflammatory bowel disease in Male Hartley albino guinea pigs. In particular, the outcome of inflammation, mucosal secretions and expression of the vasoactive intestinal peptide and substance P in the ileum and colon was analyzed. Furthermore, the effects of relaxin in counteracting observed alteration was studied. Interestingly, the ileum and colon showed a highly diverse sensitivity to cigarette smoke in regard to induced inflammatory infiltrates, fibrosis, and acidic mucin production. Moreover, relaxin reduced most of the smoke-induced changes in the ileum and was less effective in the colon, suggesting that different regions of the gastrointestinal tract are not only differently sensitive to smoke but are also differently reactive with regard to therapeutically effective substances.

The review by Verbeure et al. focusses on the role of nitric oxide (NO), carbon monoxide (CO) and hydrogen sulfide (H_2_S) on gut peptide release and functioning. The presented overview shows that these gasotransmitters have subtle roles in the regulation and function of gastrointestinal hormones including gastrin, cholecystokinin, secretin, motilin, ghrelin, glucagon-like peptide-1 and -2. As such these small molecules may be attractive therapeutic targets for pharmacological interventions in diseases associated with altered gut peptide activity. However, as the authors concluded correctly, it will be a major challenge to target them in an organ-, process-, or disease-specific manner.

Beckers et al. investigated potential associations between age-related changes in abdominal symptoms and the expression of the visceral pain-associated receptors Ankyrin one and Vanilloid in patients suffering from irritable bowel syndrome (IBS). Comparative analysis of data obtained by quantitative RT-PCR and immunofluorescence in samples of 463 IBS patients and 317 healthy controls subjects revealed significantly lower scores for abdominal pain and indigestion in both elderly IBS and healthy individuals compared to respective young adults and a lower relative expression of Ankyrin one and Vanillid in healthy elderly versus healthy young adults. This suggests that abdominal pain perception is decreased during aging, potentially providing the basis for the development of novel age-dependent pain management strategies in IBS.

The systemic review by Cheng et al. summarizes and evaluates the evidence for a pathogenetic role of the purinergic P2X7 receptor (P2X7R) in the gastrointestinal system. This receptor belongs to plasma membrane molecules found in many mammalian tissues responding to extracellular nucleotides. Based on a literature search in PubMed, embase and Scopus the authors identified 48 articles in which aspects of P2X7R during various actions under homeostatic and pathological conditions in the GI tract were analyzed. In sum, the systemic analysis of respective studies demonstrates that the activation of P2X7R can induce a broad array of cellular responses, including cytokine release, apoptosis, and cell death, all mechanisms that in the GI system significantly contribute to inflammatory processes and promotion of tumor development. However, some reports analyzed showed that P2X7R can also suppress inflammation in the course of colitits. Therefore, P2X7R antagonism might be a double-edged sword exerting beneficial and detrimental effects during the pathogenesis of IBD.

Palmitoylethanolamide is both a naturally-occurring lipid ingredient contained in foods/dietary supplements and an endogenous lipid mediator chemically-related to the endocannabinoid anandamide. D’Antongiovanni et al. provide evidence for the possible use of palmitoylethanolamide for the management of enteric inflammation and bowel dysfunctions associated with Alzheimer’s disease (AD). By using a spontaneous genetic model of AD (i.e., SAMP8 mice), the authors showed that palmitoylethanolamide, under a condition of cognitive decline, prevented the enteric glial hyperactivation, reduced AD-related proteins, counteracted colonic inflammation, improved the intestinal epithelial barrier integrity and regularized excitatory colonic contractions.

Worldwide, gastric cancer (GC) represents a common cause of morbidity and mortality in countries. The clinical impact of current therapies is modest and apoptosis evasion, i.e., one of the pivotal hallmarks of cancer. Laurino et al. demonstrated the possible mechanism by which TRPV2 pharmacological targeting with tranilast overcame cisplatin resistance (a major limitation of current therapeutic regimens for GC). This is therapeutically important because tranilast (N-3′,4′-dimethoxy cinnamoyl]-anthranilic acid), an analog of a tryptophan metabolite identified mainly as an anti-allergic agent, is already known as a safe and long-lasting drug.

The results of a retrospective study describing the use of food supplement containing flavonoids Vitamin C, and plant extracts from *Centella asiatica* (gotu kola), *Vaccinium myrtillus* (blueberry), and *Vitis vinifera* (common grape wine), prescribed from 2019 to 2020 in the hepato-gastroenterology division outpatient unit of the University of Campania “Luigi Vanvitelli” for hemorrhoid disease, were illustrated by Gravina et al. Data showed that the formulation was safe and effective in patients with grade II and III hemorrhoidal disease.

Shafrir et al. investigated the possible association between proton pump inhibitors (PPI) use and the risk of infection and development of severe disease in SARS-CoV2 infected patients. The authors used data from a health maintenance organization database in Israel that insures over 1,200,000 individuals. In a multivariable logistic regression model controlling for age, gender, smoking status, body mass index, diabetes mellitus, hypertension, chronic obstructive pulmonary disease, history of ischemic heart disease and fasting blood glucose levels, no significant association was found between PPIs and SARS-CoV-2 positivity or severe COVID-19.

## Intervention in Liver Diseases and Colorectal Cancer

The review article by Cai et al. highlights current knowledge in epigenomic alterations associated with the occurrence and progression of chronic liver disease (CLD). In particular, the authors discuss the roles of histone modifications in selected CLDs. The comprehensive overview provides information about the different forms of histone modifications and their alterations during initiation and progression of CLD and the pharmacological possibilities for targeting these epigenetic changes. The contribution highlights that histone modification can be pharmacologically modulated and that some epigenetic drugs have already been approved for clinical use. Although there are still some issues unknown, these encouraging findings might lead in the near future to the development of drugs with low toxicity and high efficacy able to improve the outcome of CLD.

The article by Chen et al. reports about the biological mechanisms by which Paeoniflorin (PF) mediates its hepatoprotective activity during cholestatic liver injury. The authors combined metabolomics and systematical network pharmacological analysis and showed that the monoterpene glycoside significantly improved liver histological damage and serum parameters indicating hepatic damage in a rat model of α-naphthylisothiocyanate-induced cholestasis liver injury model. The data provided indicates that PF evolved its protective effects on cholestatic liver injury by majorly regulating amino acid metabolisms, bile acid metabolisms and inflammation.

In the study by Cheng et al. investigated the association of nonselective-beta-blockers (NSBBs) and the incidence of hepatocellular carcinoma (HCC) in patients with chronic hepatitis B (CHB) infection without cirrhosis and decompensation. The reported findings demonstrate that NSBBs are not associated with decreased HCC occurrence suggesting that these drugs should not be recommended as a chemopreventive drugs as a way to lower HCC development in patients suffering from CHB.

In another experimental study, Du et al. evaluated the protective effects of Atractylenolide I (AO-I) against acetaminophen (APAP)-induced acute liver injury in mice. The drug was intragastrically administered 2 h before APAP dosing and histopathological changes of the liver and markers of oxidative stress and hepatic inflammation were analyzed. In their experiments, the prophylactic treatment with the sesquiterpene confers protection against APAP-induced hepatotoxicity, which can be attributed to its anti-inflammatory and anti-oxidative properties, most likely by targeting the TLR4/MAPK/NF-κB pathways.

Kositamongkol et al. systematically reviewed beneficial effects of coffee consumption and the incidence of non-alcoholic fatty liver disease (NAFLD) in the general population and on the reduction of liver fibrosis among patients suffering from NAFLD. Their bipartite study consisting of an overview of former reviews and a systematic review and meta-analysis shows that findings on therapeutic effects of coffee drinking on NAFLD prevention in the general population are contrasting, while consumption of coffee has proven beneficial effects on liver fibrosis in NAFLD patients.

Yang et al. comparatively analyzed the immunophenotypic characteristics of monocyte-derived dendritic cells in the peripheral blood of patients suffering from autoimmune hepatitis (AIH) and healthy control subjects. In their study, the authors established whole RNA-sequencing data from AIH patients and controls and used bioinformatics approaches to identify differentially expressed long noncoding RNAs (lncRNAs), circular RNAs (circRNAs), microRNAs (miRNAs), and mRNAs. The expression differences were clustered, potential interactions between lncRNA-miRNA-mRNA and circRNA-miRNA-mRNA established, and competing endogenous RNA networks between the different RNA subtypes were constructed. This fundamental study with its comprehensive data has disclosed several dysregulated RNAs associated with the pathogenesis of AIH and further provides information about new therapeutic targets for treatment of AIH.

Yang et al. performed multi-omics profiling of liver extracts and sera taken from both peripheral blood and hepatic portal vein blood from the long-tailed macaque (*Macaca facicularis*) that either spontaneously developed diabetes type 2, were fed with a normal chow diet, or received a high-fat-high-sugar diet. In this study, the authors found that the fatty acid binding protein 4 (FABP4) is a critical mediator that contributes to the pathogenesis of diet-induced and spontaneously occurring diabetes. In the liver both FABP4 mRNA and protein are significantly upregulated. In addition, the authors found that a lot of genes functioning in cell migration and cellular/extracellular structure are changed in prediabetic and diabetic animals suggesting that alterations in homeostasis of these genes preventing proper liver regeneration might promote diabetes.

In regard to insulin, another original research article authored by Zhang et al. evaluated the therapeutic effects of a water extract prepared from the Gentianaceae family member *Veratrilla baillonii Franc* (WVBF) on hepatic injury and insulin resistance in a high-fat diet- and streptozotocin-induced diabetic rat model. In their study, the authors performed liver transciptome analysis and found that the WVBF extract suppressed several genes associated with formation of insulin resistance and further reduced the pathological damage of the liver and pancreas. Combined with previous work from the group showing that the extract can attenuate hepatic toxicity, the present study provides potential explanations of the protective effects of WVBF in preventing insulin resistance and associated liver complications.

Another experimentally study authored by Zhou et al. investigated the mechanisms by which the vagus nerve activating α7 nicotinic receptors (α7nAChR) ameliorates inflammation and gastric dysmotility in Parkinson’s disease rats produced by microinjection of 6-hydroxydopamine in the substantia nigra. In their article, the authors could demonstrate that respective rats exhibit impairment of the cholinergic anti-inflammatory pathway resulting in α7nAChR-positive macrophage infiltrats in the gastric muscularis. In this context, the application of the α7nAChR inhibitor PNU-282987 alleviated gastric inflammation and improved gastric dysmotility by exerting a direct anti-inflammatory effect suggesting that drugs targeting α7nAChR might have beneficial effects on gastroparesis in Parkinson’s disease.

In the review by Singh et al. the potential of Programmed cell death protein 1/PD-ligand 1 (PD-1/PD-L1) inhibitors for the treatment of CLDs such as hepatitis, liver injury, and hepatocellular carcinoma are discussed. In particular, the article focusses on immunomodulating activities of these substances, potential therapeutic strategies resulting thereof, past and ongoing clinical studies conducted with these inhibitors, drug toxicities, and challenges associated with PD-1/PD-L1 blockade therapies. In sum, the article documents that the PD-1/PD-L1 pathway offers versatile possibilities for therapeutic intervention in chronic liver diseases, but that further studies are urgently needed to resolve existing pharmacological challenges connected with inhibitors directed against PD-1/PD-L1.

Zhang et al. provided evidence that atractyloside protects mice against high fat diet-induced liver steatosis. At molecular level, this drug blocked expression of the adenine nucleotide translocator 2 (ANT2), stimulated the activation of AMP-activated protein kinase (AMPK), decreased the activity of mechanistic target of rapamycin kinase (mTOR), and ultimately promotes autophagosomes formation, thus hastening the degradation of hepatic lipids caused by the high fat diet. Atractyloside, a diterpenoid glycoside present in a number of plant species worldwide, including *Atractylis gummifera*, is a high-affinity specific inhibitor of the mitochondrial ADP/ATP carrier, thus being able to impair energy balance.

A study by Kim et al. evaluated a newly developed plasma-based peptide nucleotide acid (PNA) probe-mediated real-time PCR kit for the detection of microsatellite instability (MSI) in 84 colorectal cancer (CRC) patients. The authors found that the PNA-based real-time PCR kit is able to detect MSI in plasma of CRC patients with a positive predictive rate of 100% and a sensitivity of about 70%. As such, the method offers an alternative diagnostic method for MSI testing in patients in whom collection of tissue samples is difficult or not available.

In sum, all these 29 articles show that the different gastrointestinal and hepatic diseases are complex disorders. Although there has been great success in understanding the pathogenesis of the different disease aetiologies, there is still an urgent need to identify suitable druggable targets. The collection of articles published in this Research Topic shows that the possible repertoire of respective targets is extremely large. In addition, preclinical work from the different laboratories has identified already several substances or mixtures showing highly beneficial effects in the setting of a specific gastrointestinal or hepatic disorders. However, there remains a lack of translating respective findings into clinical studies or novel therapies and future studies are warranted before effective therapeutics can be developed for clinical use.

